# Evaluation of the Potential for Improvement of Clinical Outcomes in Trauma Patients with Massive Hemorrhage by Maintaining a High Plasma-to-Red Blood Cell Ratio during the First Hour of Hospitalization

**DOI:** 10.1155/2023/5588707

**Published:** 2023-07-18

**Authors:** Junsik Kwon, Jayoung Yoo, Sora Kim, Kyoungwon Jung, In Kyong Yi

**Affiliations:** ^1^Division of Trauma Surgery, Department of Surgery, Ajou University School of Medicine, 164 Worldcup-ro, Yeongtong-gu, Suwon-si 16499, Gyeonggi-do, Republic of Korea; ^2^Department of Anesthesiology and Pain Medicine, Ajou University School of Medicine, 164 Worldcup-ro, Yeongtong-gu, Suwon-si 16499, Gyeonggi-do, Republic of Korea

## Abstract

Several reports indicate that early plasma transfusion may promote survival and reduce the incidence of traumatic coagulopathy in situations of massive bleeding. Consequently, it is recommended to maintain a plasma and RBC transfusion ratio between 1 : 1 and 1 : 2 at the start of admission. This retrospective study examined the effect of an early high plasma : RBC ratio on mortality rates by adopting a massive transfusion protocol (MTP) that forced an early and rapid issue of plasma products. Patients who received massive transfusions at a single trauma center between January 2014 and May 2020 were included in the study. A new protocol was established in January 2020, wherein a fixed amount of plasma was issued following MTP activation. Patients who underwent massive transfusions before and after the adoption of the new protocol were compared. In total, 1059 patients met the inclusion criteria. Fifty-one patients who underwent MTP were propensity score-matched with the patients who received a nonprotocolized massive transfusion. The MTP group had a higher plasma : RBC ratio at 1 h (0.8 vs. 0.2) and 4 h of hospitalization (1.1 vs. 0.6), with no significant between-group difference in the plasma : RBC ratio at 24 h of hospitalization. The MTP group had a lower 24 h mortality rate than the control group. There was no significant difference in the 30-day mortality. Using MTP to achieve a high plasma : RBC ratio in the early period of hospitalization appeared to affect 24-hour mortality; however, 30-day mortality did not change.

## 1. Introduction

Massive transfusion is defined as follows: (1) the replacement of the total blood volume in 24 h, (2) the replacement of 50% of the blood volume in 3 h, or (3) an emergency transfusion for a clear case of critical bleeding. Critical bleeding refers to when at least 4 units of red blood cells (RBCs) are transfused within 4 h or blood is transfused at 150 mL/min in an adult [1]. Several reports suggest that early transfusion of plasma and platelets can benefit survival and reduce the incidence of traumatic coagulopathy in massive bleeding situations [2–5]. A Pragmatic, Randomized Optimal Platelet, and Plasma Ratio (PROPPR) study achieved rapid hemostasis and fewer deaths attributed to exsanguination [6]. Based on the results of this study, the Trauma Quality Improvement Program (TQIP) of the American College of Surgeons (ACS) recommends maintaining a plasma and RBC transfusion ratio between 1 : 1 and 1 : 2 at the beginning of admission [7]. However, since plasma is usually stored in a frozen state, its prompt administration similar to that of RBC is difficult; hence, administering enough plasma at the beginning of hospitalization during traumatic coagulopathy is considerably challenging.

Thus, this study aimed to achieve a high plasma-to-RBC ratio in the early phase of massive transfusion using a newly updated massive transfusion protocol (MTP) with a quick issue of plasma products at a level-I trauma center. Furthermore, we aimed to compare the survival benefit of patients who received transfusions using the new MTP with that of patients who received transfusions according to the previous protocol.

This study was approved by the Institutional Review Board of Ajou University (AJIRB-MED-MDB-21-373), and the requirement for patient consent was waived owing to the retrospective study design.

## 2. Materials and Methods

Adult patients aged ≥15 years hospitalized at the Ajou University Trauma Center between January 2014 and May 2020 who received a massive transfusion were included in the study. The Ajou University Trauma Center is a level-I trauma center designated by a governmental health organization that covers approximately 9 million people across the South Gyeonggi Province, South Korea. The center admits approximately 2,000 patients every year, and 45% of all the patients admitted to the center have an injury severity score (ISS) of ≥16 points.

Massive transfusion was defined as a transfusion of ≥10 units of RBC in the first 24 h of hospitalization or 4 units of RBC in the first 4 h of hospitalization [1, 7]. The protocol used previously was merely a “recommendation” for transfusing plasma as promptly as possible for the maximum number of RBCs used (Supplement 1). However, it was not efficient since the composition and quantity of each MTP batch were randomly determined each time. This was the main cause of the logistic problem, resulting in the plasma delivery delay. The protocol updated in January 2020 forced a fixed ratio of plasma to be drawn from a blood bank according to the RBC prescription amount in the MTP situation. Moreover, the composition of the batch was decided for an amount that could be promptly thawed and provided in consensus with the blood bank (Supplement 2). Demographic and clinical data were obtained from electronic medical records, which were collected by the Korean Trauma Data Registry and the hospital. All trauma deaths were reviewed by a mortality review committee operating for quality improvement, and the “types of death” were determined according to the results of the meeting.

### 2.1. Statistical Analysis

All the statistical analyses were performed using the Statistical Package for Social Sciences (SPSS) v24. Independent *t*-tests were performed to measure the differences between continuous variables, and a chi-square test was used to measure the differences between categorical variables. Kaplan–Meier curves were used for the survival analysis, and *p* < 0.05 was considered statistically significant. The control group was matched with the MTP group based on propensity scores in a 1 : 1 ratio using age, sex, initial systolic blood pressure, and ISS (Injury Severity Score). The matching tolerance was set to 0.01 to minimize the difference between the matched control group and the MTP group. Additional analyses, including mortality analysis, were performed using the control group thus created.

## 3. Results

**Figure 1:**
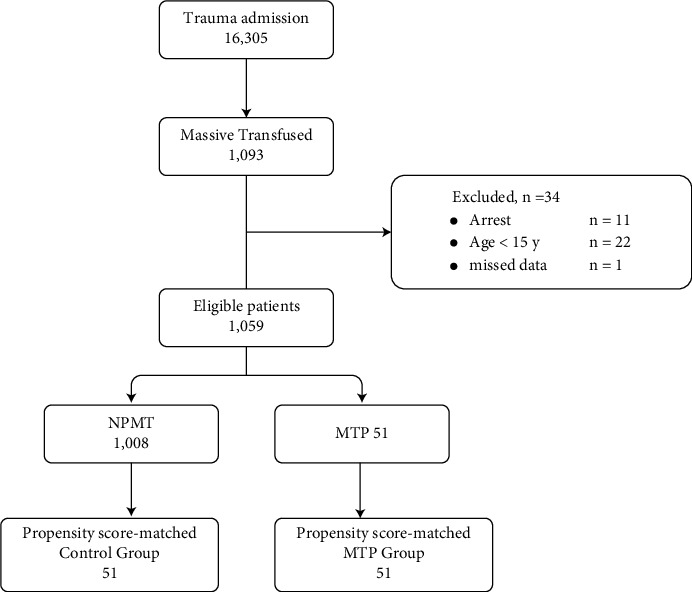
Flowchart of patient inclusion.

A total of 16,305 patients were admitted to the level-I trauma center and enrolled in the Korean Trauma Data Bank from January 2014 to May 2020. Of these, 1,093 patients who received massive transfusions were enrolled in this study. We excluded 11 patients who were considered “dead on arrival” with a heart rate of 0, systolic blood pressure of 0, and Glasgow coma scale score motor component of 1 [8]. In addition, 22 patients aged ≤14 years and one patient whose transfusion amount was not precisely recorded was excluded. Finally, the remaining 1,059 patients were deemed eligible and included in this study (Figure 1).

Table 1 presents the general characteristics of the 51 patients who received MTP and the 1,008 patients who received nonprotocolized massive transfusion (NPMT). The MTP group was younger and had a lower initial blood pressure than the NPMT group. The MTP group also had a higher percentage of patients with the assessment of blood consumption scores ≥2 points, a higher ISS, and a higher mean score for extremity on the abbreviated injury scale (AIS) than did the NPMT group (Table 1).

The NPMT group was matched with the MTP group based on propensity scores in a 1 : 1 ratio using age, sex, initial systolic blood pressure, and ISS. After matching, we found no significant differences between the two groups in most variables, including hemoglobin level, for which differences were observed before matching. Differences in the mean scores for extremities on the AIS remained after matching (Table 2).

In a comparison of the initial blood transfusion time and the transfusion amount and ratio over time, the mean initial fresh frozen plasma (FFP) transfusion time was 57.5 minutes shorter in the MTP group than in the control group; moreover, the MTP group demonstrated significantly higher RBC and FFP transfusion amounts at 1 h of hospitalization (Table 3).

**Figure 2:**
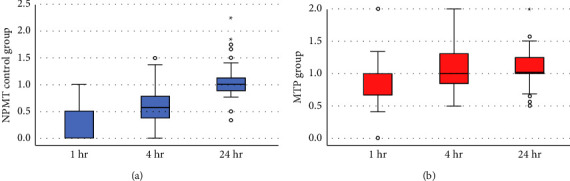
Plasma-to-RBC ratio at 1 h, 4 h, and 24 h.

The MTP group had a higher FFP-to-RBC ratio at 1 h of hospitalization than the control group (0.8 vs. 0.2). The group also had a higher FFP-to-RBC ratio in the first 4 h of hospitalization than the control group (1.1 vs. 0.6). No difference was found in the FFP-to-RBC ratio in the first 24 h of hospitalization between the two groups (Figure 2).

During the first 1 h and at 4 h, the plasma-to-RBC ratio was low, and the cumulative ratio at 24 h reached 1 : 1 in the NPMT control group, whereas in the MTP group, the plasma-to-RBC ratio was maintained at 1 : 1 from the first 1 h, at 4 h, and until 24 h.

NPMT: nonprotocolized massive transfusion; MTP: massive transfusion protocol; and RBC: red blood cell.

**Figure 3:**
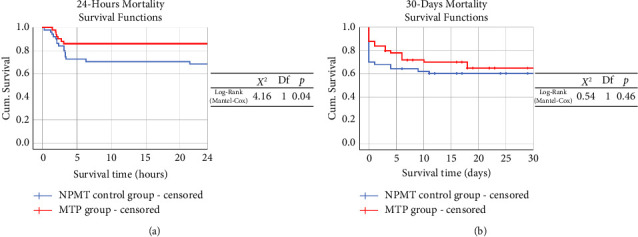
Kaplan–Meier survival curve.

In the comparison of the outcomes, the MTP group had a significantly lower 24 h mortality rate than the control group. No differences were found based on the 30-day mortality, other outcomes, and procedures for hemostasis, including surgery and angioembolization, between the two groups (Table 4, Figure 3).

The 30-day mortality was not significantly different; however, the 24 h mortality was significantly lower in the MTP group than in the NPMT group.

MTP: massive transfusion protocol and NPMT: nonprotocolized massive transfusion.

## 4. Discussion

Several studies dealing with transfusion strategies for patients with massive bleeding trauma report the advantages of the fixed-ratio transfusion strategy. Thus, many trauma centers have adopted an MTP using a 1 : 1 transfusion ratio. However, observational studies examining the effect of a high plasma-to-RBC ratio in trauma patients are subject to survivor bias since plasma administration generally starts after RBC administration [9]. Survivor bias or survivorship bias is a type of selection bias based on the logical error of concentrating on the people or things that made it past a selection process and overlooking those that did not, typically owing to their lack of visibility. Based on the survivor bias, the results can be interpreted as having an association between high plasma administration and a high survival rate merely because the survivors had the opportunity to receive more plasma. In a study of 806 patients analyzed using logistic regression, Scalea et al. [10] demonstrated that a high plasma-to-RBC ratio had no benefits on survival except in cases of early death. However, the duration of intensive care unit hospitalization was not investigated, and the mortality rate in the massive transfusion group was too low (6%). A review of studies on massive transfusion performed on trauma patients from 1966 to 2011 reported that 11 of the 26 included articles were prone to survival bias [11]. Many studies on trauma resuscitation reported that most of the early deaths were due to bleeding within 2-3 h of hospitalization [2, 5, 12–14]. Therefore, to examine the effect of early plasma administration via a fixed-ratio transfusion without survival bias, the plasma-to-RBC transfusion ratio should be examined at 2-3 h rather than at 24 h of hospitalization. Therefore, to circumvent the risk of survivorship bias, the MTP group (which received sufficient plasma during the first hour of the patient's admission) was set as the study group.

In this study, the MTP using a fixed-ratio transfusion strategy maintained a stable plasma-to-RBC ratio in the first 1 and 4 h of hospitalization. Before the adoption of the fixed-ratio transfusion strategy, most plasma transfusions were administered after the first 4 h of hospitalization, thus, failing to reach the 1 : 1 ratio in the first 1–4 h of hospitalization. Although both groups reached a plasma-to-RBC ratio of 1 : 1 after 24 h of hospitalization, the fixed-ratio transfusion group had lower 24 h mortality, while several studies report the benefit of a high plasma-to-RBC ratio in 12- and 24-hour survival rates following trauma [1, 9], others do not report a 30-day survival benefit [8]. No statistical difference was observed in the 30-day mortality in this study. Although the “PROPPR study,” a large-scale, multicentric randomized clinical trial, did not report the overall mortality benefit of the strategy of using a high plasma-to-RBC ratio, the reduction in mortality due to exsanguination was also noted in its results [10].

There is a lack of conclusive studies on optimal plasma-to-RBC ratios and specific patient subgroups for which early plasma administration may provide clear benefits. However, early plasma transfusion has been reported to reduce trauma-induced coagulopathy and systemic endothelial damage caused by traumatic hemorrhage [10, 11]. This strategy is effective against hyperfibrinolysis, one of the most lethal phenotypes of trauma-induced coagulopathy [12]. Large-scale studies on the MTP for patients with severe traumas, such as “PROMMT [5]” and “PROPPR [6],” clearly demonstrated the importance of early plasma administration. However, there are obstacles to early plasma administration, such as logistical problems related to universal donor AB plasma, the short expiry date of thawed plasma, and a potential increase in the blood disposal rate [15]. These obstacles hinder early plasma administration in many trauma centers that use their own protocols adapted from an MTP.

In the trauma center where this study was conducted, frequent delays in FFP delivery were discovered in the quality improvement activities, which was the reason for initiating the work for updating the previous transfusion protocol. This study was conducted to implement such a quality improvement program. The trauma center in which this study was conducted computerized the blood application process after updating the MTP and determined the optimal amount of FFP to avoid thawing delays and deliver promptly upon request. Furthermore, the blood bank, nursing department, blood transportation workforce, and the quality improvement team of the trauma center worked together to improve the blood-issuing processes. The initial time of administration of the type-matched FFP was significantly shortened. This study achieved a fixed-ratio transfusion in the early period of hospitalization merely by improving the logistics of the type-matched plasma-issuing process without the use of thawed universal plasma and significantly improved the mortality rates.

Our study has some limitations. First, we found no improvement in platelet administration, which is another major blood product used in the 1 : 1 : 1 balanced transfusion. Batch administration was not possible based on the number of transfused RBCs, due to insufficient storage of the platelet concentrates. Second, all types of deaths were included in the mortality analysis, and deaths attributable to causes other than hemorrhage, such as severe brain trauma, could not be excluded. As excessive bleeding leads to sudden death from a haemorrhagic shock or death from multiorgan failure resulting in an irreversible ischemic insult on multiple vital organs; hence, bleeding cannot be completely excluded as the cause of death. Third, due to the retrospective nature of the study, we could not control the effects of other variables, excluding the implementation of the improved MTP using a fixed-ratio transfusion, on the study and control groups. To overcome this limitation, propensity score-matching using early vital signs and other variables related to trauma intensity was used. However, the small difference in AIS scores at extremity was not statistically fully adjusted. Finally, the control and experimental groups in this study were not collected at the same time. In the meantime, there have been several medical advances, so the improvement in outcomes may not all be due to the effect of the revised MTP.

## 5. Conclusions

In conclusion, we updated the MTP to maintain plasma levels similar to RBCs in the first and fourth hours, which did not lower the 30-day mortality rate in trauma patients requiring massive transfusions but did appear to affect 24-hour mortality.

## Figures and Tables

**Table 1:** General characteristics of each group (*N* = 1,059).

	NPMT (*n* = 1008)	MTP (*n* = 51)	*p*
Age (years, mean)	51	45	**0.017**
Male	773 (76.7%)	35 (68.6%)	0.187
Glasgow coma scale score (mean)	9.2	9.7	0.477
Systolic blood pressure			
Mean (mmHg)	101	74	**<0.001**
*n* with ≤90 mmHg	377 (37.4%)	30 (58.8%)	**0.002**
Diastolic blood pressure			
Mean (mmHg)	64	49	**0.005**
Heart rate (*n*)	919	47	
Mean (beats/min)	102	108	0.115
*n* with ≥120 beats/min	241 (26.2%)	12 (25.5%)	0.916
Assessment of blood consumption score (*n*)	680	43	
*n* with ≥2	150 (22.1%)	13 (30.2%)	0.214
Focused assessment with sonography in trauma (*n*)	734	45	
Positive	194 (26.6%)	12 (26.7%)	0.972
Mechanism of injury			
Blunt injury	924	47	0.902
Penetrating injury	84	4	
Hemoglobin level (*n*)	967	50	
Mean (g/dL)	10.8	11.6	**0.006**
*n* with ≤11 g/dL	494 (51.1%)	18 (36.0%)	**0.037**
International normalized ratio (*n*)	929	47	
Mean	1.4	1.6	0.088
*n* with ratio >1.5	210 (22.6%)	14 (29.8%)	0.253
Lactic acid (*n*)	976	51	
Mean (mmol/L)	6.0	6.9	0.128
*n* with ≥2.0 mmol/L	910 (93.2%)	50 (98.0%)	0.176
Base excess (*n*)	962	51	
Mean (mmol/L)	−9.0	−9.9	0.279
*n* with score ≤ −4 mmol/L	793 (82.4%)	46 (90.2%)	0.152
Injury severity score (*n*)	969	51	
Mean	28.9	37.1	**0.002**
AIS head (*n*)	462	25	
Mean	3.9	3.5	0.177
AIS face (*n*)	239	11	
Mean	1.8	2.0	0.401
AIS chest (*n*)	239	11	
Mean	3.2	3.4	0.178
AIS abdomen (*n*)	663	40	
Mean	3.2	3.1	0.594
AIS extremity (*n*)	677	39	
Mean	3.0	3.7	**0.002**
AIS external (*n*)	248	24	
Mean	1.1	1.1	0.546

AIS: abbreviated injury scale; NPMT: nonprotocolised massive transfusion; MTP: massive transfusion protocol. *P* values less than 0.05 were indicated in bold.

**Table 2:** General characteristics of the propensity score-matched patients (*n* = 102).

	NPMT-matched control (*n* = 51)	MTP (*n* = 51)	*p*
Age (years, mean)	45	45	0.913
Male (*n* (%))	39 (76.5)	35 (68.6)	0.375
Glasgow coma scale score (mean)	9.5	9.7	0.353
Systolic blood pressure (*N* (51/51))			
Mean (mmHg)	69	74	0.523
No. (%) with ≤90 mmHg	34 (66.7)	30 (58.8)	0.413
Diastolic blood pressure (*N* (51/51))			
Mean (mmHg)	41	49	0.275
Heart rate (*N* (37/47))			
Mean (beats/min)	105	108	0.538
*n* (%) with ≥120 beats/min	10 (27.0)	12 (25.5)	0.878
Assessment of blood consumption score (*N* (32/43))			
No. of ≥2 (%)	12 (37.5)	13 (30.2)	0.509
Focused assessment with sonography in trauma (*N* (40/45))			
*n* (%) with positive of ≥2	14 (35.0)	12 (26.7)	0.405
Mechanism of injury (*n*)			0.727
Blunt injury	46	47	
Penetrating injury	5	4	
Hemoglobin level			
Mean (g/dL)	10.8	11.6	0.143
International normalized ratio			
Mean	1.5	1.6	0.593
Lactic acid			
Mean (mmol/L)	7.3	6.9	0.647
Base excess			
Mean (mmol/L)	−11.4	−9.9	0.256
Injury severity score			
Mean	35.1	37.1	0.548
AIS head			
Mean	3.78	3.52	0.489
AIS face			
Mean	1.67	2.00	0.228
AIS chest			
Mean	3.58	3.38	0.364
AIS abdomen			
Mean	3.16	3.09	0.781
AIS extremity			
Mean	3.06	3.67	**0.035**
AIS external			
Mean	1.14	1.08	0.655
Pre-hospital time			
Mean	33.4	35.3	0.490

AIS: abbreviated injury scale; NPMT: nonprotocolised massive transfusion; MTP: massive transfusion protocol. *P* values less than 0.05 were indicated in bold.

**Table 3:** Transfusion time and plasma-to-RBC ratio (*n* = 102).

	NPMT-matched control (*n* = 51)	MTP (*n* = 51)	*p*
Initial RBC transfusion time (min)	13.7	12.1	**0.028**
Initial plasma transfusion time (min)	72.7	15.2	**0.001**
Transfused units of RBC in the first 1 h	5.0	7.1	**0.005**
Transfused units of plasma in the first 1 h	0.8	5.4	**<0.001**
Transfused units of RBC in the first 4 h	10.1	11.9	0.180
Transfused units of plasma in the first 4 h	5.5	11.9	**<0.001**
Transfused units of RBC in the first 24 h	14.0	15.8	0.550
Transfused units of plasma in the first 24 h	13.5	17.8	0.338
Plasma: RBC ratio in the first 1 h	0.2	0.8	**<0.001**
Plasma: RBC ratio in the first 4 h	0.6	1.1	**<0.001**
Plasma: RBC ratio in the first 24 h	1.0	1.1	0.146

AIS: abbreviated injury scale; NPMT: nonprotocolised massive transfusion; MTP: massive transfusion protocol RBC: red blood cells. *P* values less than 0.05 were indicated in bold.

**Table 4:** Outcomes of each group (*n* = 102).

	NPMT-matched control (*n* = 51)	MTP (*n* = 51)	*p*
24 h mortality, *n* (%)	16 (31.4)	7 (13.7)	**0.03**
30-day mortality, *n* (%)	20 (39.2)	17 (33.3)	0.54
Ventilator day, mean	13.0	8.9	0.339
ICU day, mean	17.8	10.9	0.087
Hospital day, mean	47.7	31.3	0.058
Ventilator-associated pneumonia, *n* (%)	8 (15.7)	5 (9.8)	0.37
Operation for hemostasis, *n* (%)	24 (47.1)	24 (47.1)	0.93
Radiologic intervention for hemostasis, *n* (%)	3 (5.9)	6 (11.8)	0.30

ICU, intensive care unit; NPMT: nonprotocolised massive transfusion; MTP: massive transfusion protocol. *P* values less than 0.05 were indicated in bold.

## Data Availability

The datasets used and/or analyzed during the current study are available from the corresponding author on reasonable request.

## Supplementary Materials

Supplementary Materials Supplement files were attached for detailed comparison of the existing massive transfusion protocol and the changes.
