# Supplementary material for: Evaluation of the Potential for Improvement of Clinical Outcomes in Trauma Patients with Massive Hemorrhage by Maintaining a High Plasma-to-Red Blood Cell Ratio during the First Hour of Hospitalization
Source: Emerg Med Int. 2023 Jul 18;2023:5588707. doi: 10.1155/2023/5588707 (PMC10368501; doi:10.1155/2023/5588707)
Supplement: Supplementary Materials — Supplement files were attached for detailed comparison of the existing massive transfusion protocol and the changes. [file 5588707.f1.zip › supplement 1.docx]

**Ajou Massive Transfusion Protocol (MTP)**

1) Trauma leader decides whether to activate MTP considering the following after trauma team activation.

A. Mechanism of injury and physiological indicators

B. Coagulopathy and taking anticoagulants

C. Hemodynamic instability criteria: 3 or more of the following 6 items

(1) Heart rate (HR) > 110/min

(2) Systolic blood pressure (SBP) < 100 mmHg

(3) Hemoglobin (Hgb) < 9 g/dL

(4) pH < 7.25

(5) Lactic acid > 4.0 mmol/L

(6) Body temperature < 35 ℃

2) If MTP is required, emergency transfusion of O + pRBC 6 units in trauma bay is performed.

3) When the MTP is activated, insert one or more MAC catheters, actively use the rapid infusion system, and notify the intensive care unit and operating room in advance to prevent blood transfusion from being interrupted.

4) Apply for emergency transfusion of O+ pRBC 6 units or Unmatched type-specific pRBC 6 units / type specific fresh frozen plasma (FFP) 6 units while sending to the blood bank after taking blood for blood preparation.

5) According to the criteria corresponding to 1), decide whether to continue the MTP once more.

6) MTP continuation has been determined. Request for rapid transfusion of Crossmatched type-specific pRBC 6 units / FFP 6 units. If necessary, mix with fluid for transfusion, but follow up the patient's condition and lab findings and follow the leader's decision. In this case, use Normal saline or Plasma solution A for the sap.

7) Determine whether to continue MTP by collecting blood for ABGA, lactic acid, and CBC at least once every 3–4 hours.

8) In accordance with the principle of Damage Control Resuscitation, adjust the amount of FFP and platelet transfusion so that pRBC : FFP : platelet = 1 : 1 : 1 in the shortest possible time (24 hours).
