# Supplementary figures and images for: Evaluation of the Potential for Improvement of Clinical Outcomes in Trauma Patients with Massive Hemorrhage by Maintaining a High Plasma-to-Red Blood Cell Ratio during the First Hour of Hospitalization
Source: Emerg Med Int. 2023 Jul 18;2023:5588707. doi: 10.1155/2023/5588707 (PMC10368501; doi:10.1155/2023/5588707)

Supplement 2 Ajou Trauma Center Massive Transfusion Protocol


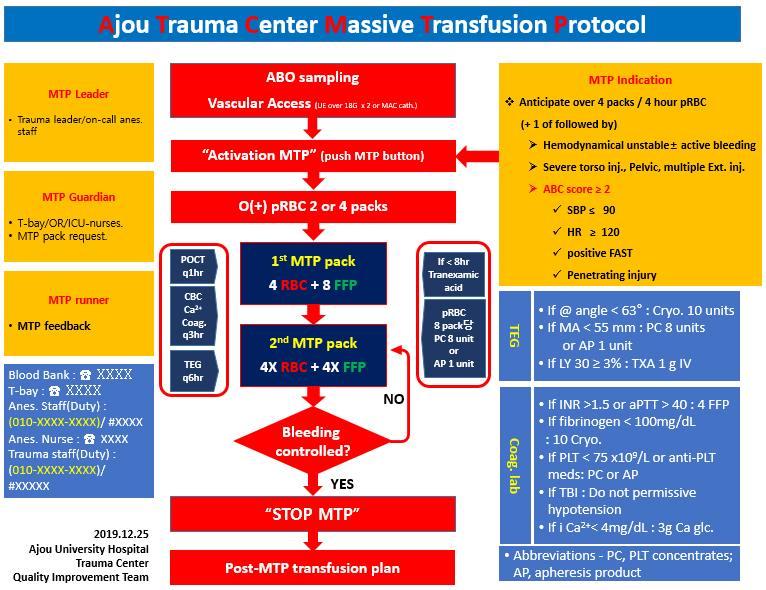

Supplement: Supplementary Materials — Supplement files were attached for detailed comparison of the existing massive transfusion protocol and the changes. [file 5588707.f1.zip › Supplement2.docx]
